# 

**Published:** 1812-12

**Authors:** 


### MONTHLY CATALOGUE OF MEDICAL BOOKS.

THE First Lines of the Practice of Surgery, designed as an introduction for Students, and a concise book of reference for Practitioners; with copper plates. The Third Edition, thoroughly revised, carefully corrected, and considerably enlarged. By Samuel Cooper. 8vo.—Longman and Co.

An Account of a Case of Recovery after an extraordinary Accident, by which the Shaft of a Chaise had been forced through the Thorax. By William Maiden. 4to.—Rivington.

An Essay on Diet and Regimen, as indispensable to the recovery and enjoyment of firm health, especially to the indolent, studious, delicate, and invalid; with appropriate cases. By J. M. Adair, M.D. 8vo.—Ridgway.

An Inquiry into the Structure and Animal Economy of the Horse, comprehending the Diseases to which his Limbs and Feet are subject, with proper directions for Shoeing; illustrated with seventeen copper plates. By Richard Lawrence. Royal 8vo.—Baldwin.

The Morbid Anatomy of the Liver; being an Inquiry into the Anatomical Character, Symptoms, and Treatment, of certain Diseases, which impair or destroy the Structure of that Viscus. Order I. Tumors.—Part the 2d on the Tubera Circumscripta, and Tubera Dissura. Imperial 4to., with colored Engravings. Part I. By J. R. Farre, M.D.—Longman and Co.

---

### *NOTICES TO CORRESPONDENTS.*

*Mr. Bucknell's communication was necessarily deferred, from our great press of matter this month.*

*We have the pleasure to announce that it is the intention of Mr. Blair, surgeon of the Lock Hospital, to communicate the substance of his observations and experience on the Venereal Disease, in the successive Numbers of this Journal. Mr. Blair is said to have entirely satisfied himself that the common opinion of Syphilis being imported from America by Columbus, is totally erroneous; and that he has arrived at this conclusion, after examining all the evidence of Astruc, Girtanner, and other celebrated writers, by whom the American origin is supported. Our readers will recollect that we formerly noticed the delivery of an oration upon this subject by Mr Blair, at the Anniversary Meeting of the Medical Society of London. (See Med. and Phys. Jour. vol. 25, p. 364.)*

*The Editors of this Journal, desirous to make known as early as possible the contents of all works of merit upon professional subjects, request that authors who wish their publications should be noticed, will direct a copy to be sent to the printer or publisher of this Journal, addressed to the Editors.*

---

### *CORRIGENDUM.*

In our last, p. 422, for *Rev. J. Nightingale and Rev. J. Andrewes, Curators*, read *W. C. Pettigrew and J. Andrewes, Esquires.*

### INDEX

### TO THE TWENTY-EIGHTH VOLUME.

ANATOMY, morbid, observations on 2, 12
———— of the brain, by Dr. Ramsay 12
Ammonia, carbonas, in scrophula 17
*Arsenic*, on tests for 66, 70, 109, 187, 284
Amputation, its fatal termination in the navy 89
———— its causes 91
————, method of preventing 92
Arsenic, on the effects of, by Mr. Brodie 117
Antimony, tartrite of, experiments with 126
Arsenic, cure for the bite of snakes in the West Indies 156
*Atropa Belladonna*, analysis of, by Vauquelin 205
Apoplexy, anatomy of 212
————, state of the brain in 214
————, causes of 216
————, symptoms of 217
————, treatment of 218
Alcyonia, in the Isle of Wight, observations on 237
Aneurism, notices of important operations for 248
*Atropa Belladonna*, its effects on the eye 258
Ammoniaco-nitrate of silver, recipe for 286
Anatomy and physiology of plants 319
Arsenic, cases of poisoning by 345
Amputation, French unsuccessful in 370
Apoplexy, observations on bleeding in, with cases 371, 2, 3, 4, 5
Apothecary, observations on his avocation 403, 412
———— meetings for reform of 404, 412
Atmosphere, pressure of, remarkable facts on 420
*Ash*, Dr. Harveian Oration 423
Alkaline salt, patent for separating from various acids, by Mr. Hodson 425
Arterial system, varieties in, by Mr. Jukes (with a plate) 440
Aphrodisiacs, M. Cossigny's 475
Arteries, unusual conformation of 505
Africa, interior of, notice of a projected journey to 515
*Bionomia* of Dr. Buchan, observations on 13
Baths of sea-water, projected establishment of in London 26
Bills of mortality for Portsmouth, by Dr. Spalding 32
Blood, experiments on the serum of 67
Bile, remarks on its use in the system 98
*Brodie*, Mr. on the action of poisons 115
Barites, muriate of, experiments with 123
Botanical Report 163, 340
Bister, &c. on their analogy with the native bitumens 235
Barbadoes, structure of 238
————, late phenomenon at, account of 251
*Belladonna*, its effect when applied on the palpebræ 258
*Bampfield*, Mr. on diseases in the ship Belliqueux 269
BOOKS, NEW MEDICAL, &c. CRITICAL ANALYSIS OF.—Medico-Chirurgical Transactions, Vol. II. 56, 151. Pharmacologia, by Dr. Paris, 69. Narrative of a Case of Cancer, 72. Edinburgh Med. and Surg. Journal, 139, 221. Dr. Cheyne's Cases of Apoplexy and Lethargy, 211. Experimental Examination of the London Pharmacopœia, by R. Phillips, 226. History of the Royal Society, by Dr. Thomson, 314. Principles of Physiological and Physical Science, by R. Saumarez, esq. 330, 414. Pamphlets on the Profession of the Apothecary, 403. Chamberlaine Treatise on Dolichos Pruriens, 413. Dr. Pitta on the Influence of Climate, &c. 488 Edin. Medical and Physical Journal, No. XXXI. 494.
Botany, origin and progress of 318
Bleeding, successful employment of in hydrophobia 352, 438
Blood-vessels, on the compression of, in diseases 368
Blood, researches on, by Brande 386
————, on the serum of 391
————, experiments on the coagulum of 339

Blood, on the coloring matter of 394
——, coloring matter of, independent of iron 401
Books published in America, catalogue of 432
*Boscovich*, his theory of fluidity 434
*Black*, Dr. ditto 435
*Bampfield*, Mr. on bleeding in hydrophobia 438
*Besnard*, M. anti-syphilitic tincture, composition of 473
Blood-letting in typhus 476
*Blair*, Mr. notice of his intended series of communications to this Journal, on Syphilis 520
Cases—Two of gutta serena, from cancer, 28; inflammation of the spinal marrow, 30; of aneurism, 57: of hernia cerebri, 59, with a plate, 186; wound of the heart, 59; of elephantiasis, with anatomical examination of the skin, 60; of poisoning by arsenic, 63; of poisoning with opium, 80; ol. terebinth, in vermes, 81; ossification of the semilunar valves of the aorta, 82; hernia umbilicalis operated on during gestation, 83; of affection of the brain, 86; small-pox after vaccination, 111; of hydrothorax, 133; of vermes cured by ol. tereb., 135; of scirrhous stomach, 139; of dysphagia, 151; of trismus, 154; of poisoning by external application of hydrarg. oxymurias, 223; of palsy cured by titillation, 224; rupture of the urethra, 245; of ulcers in the duodenum, 247; of cataract from external injury, 264; of poisoning by arsenic, 345; of hydrophobia, 351; successfully treated, 352; of cataract, 364; of apoplexy, 372; tumors in the thorax, 381; of remarkable vaccination, 382; of morbid sensation, 443; of hydrophobia, 449; of paralysis, 453; of secondary small-pox, 456; of urinary calculus, 496; of lythotomy, ib.; of ischuria vesicalis after lythotomy, 501; of encysted tumor of the brain, with dissection, 502; of painful subcutaneous tubercle, 507.
Calculus, remedy for, by Sir John Sinclair 36
Collectanea Medica, &c. 42, 115, 193, 304, 386, 470
Caoutchoc, nat. hist. and properties of 52, 107
Catalogue of professors and students at Fairfield 79
Catalogue, monthly, of books 88, 168, 256, 344, 431, 520
Clarke, Dr. on ipecacuanha enema in dysentery 93
Cancer, on arsenic in 141
———, occult, case of 142
Catalepsy, cure of, by Dr. Wilmer 169
———, observations on 376
Carbonic oxide and caloric, on a gaseous compound of, by Mr. John Davy 195
Conversions of functions or structure, remarks on 220
Chamberlaine, Mr. on the new Medicine-act 233
Cataract, Mr. Stevenson on 257, 358
———, observations on Mr. Saunders' operation for 260
———, case of, from accidental puncture of the eye 264
*Charnock*, Mr. on purgatives in typhus 278
Crocus sativus, remarks on 320
Chemistry, papers on, in the Phil. Trans. 327
———, British school of 327
Colorification in general, remarks on 335
Chyle, composition of 387
Combustion, spontaneous, instances of 514
Crocodile, fossil 516
*Callow*, his general catalogue 516
Dysentery, on the treatment of 33, 67
Droitwich, account of the brine springs at 74
Dysentery, ipecacuanha enema in 93
*Duncan*, Dr. on a soporific from *lactuca sativa* 130
*Davy*, Sir H. on phosphorous and sulphuric acids 157
Deaf and dumb, on the art of teaching to speak 193
Diseases, table of, on-board the Belliqueux 269
*Davis*, Mr. cases of poisoning by arsenic 345
*Davy*, Sir H. observations on a passage in his elements 357
Druggists, mischiefs arising from their prescribing 405
*Dickson*, Dr. on the use of purgatives 446
*Eupatorium perfoliatum*, observations on 15, 476
Elastic gum, nat. hist. and properties of 52, 107
Earthquakes in South America and West Indies 161

Electrical machines, description of 265

Equilibrium of liquids and solids 415

Emetics, useful in a case of paralysis 453

Fever, remarkable, at Blackaton, on Dartmoor 23

*Fogo*, Mr. on chronic hepatitis 95

Fever at Paisley, history of 131

——- of Sicily, history of 221

*Ferrum tartarizatium*, composition and use of a new solution of, by Dr. Birkbeck 244

Fever, observations on the theories of 294

——-, cold affusion in 296

——-, calomel and antimony in 297

*Forster*, Mr. history of tumors in the thorax 381

*Farre*, Dr. remonstrance to Mr. Stevenson 467

*Garthshore*, Dr. biography of 42

*Grew*, Dr. N. short biography of 319

Gassification, on the process of 414

Gravity and levity of liquids and solids 417

———— of gases 417

*Home*, on middle lobe of the prostate gland 6

Hernia, remarks on, by Mr. Taunton 39

Hepatitis, chronic, observations on 95

*Hume*, Mr. on his test for arsenic 109, 284

Hernia cerebri, (with a plate) 186

Hippurites of Sicily, remarks on 238

Hydrophobia, observations on 249

*Harrup*, Mr. on electrical machines 265

Hydrophobia, on the treatment of, by blood-letting 270

————, its resemblance to gastrites 275

Hepatitis, remarks on 279

*Hume*, Mr. reply to Dr. Roget 284

Hepatitis, chronic, observations on 300

Hydrophobia, case of 350

————, successfully treated 352

Hydrocele, radical cure for 424

Hydrophobia, case of, by Dr. Pinckard 449

*Hamilton*, Mr. on retarded appearance of the vac. vesicle 452

*Harrold*, Mr. case of paralysis 453

Head, forms of, the criterea of intellect 432

*Hebb*, Mr. notice of his translation of Corvisart, in diseases of the heart 517

Intestines, on wounds of, by Mr. Travers 8

INTELLIGENCE, medical and philosophical 73, 157, 233, 338, 422, 512

Intus-susceptio, on 129

*Jukes*, Mr. on some varieties in the arterial system 44[illegible]

*Kinglake*, Dr. observations on hydrophobia 273

————, on morbid sensation 441

Liver, remarks on its organic changes 4

Letters on the study of medicine 26

Licentiates, observations on their rights 49

*Labrador*, geology of 73

*Litle*, Mr. on the fatal termination of wounds in the navy 89

————, the probable causes of 90

————, method of preventing 92

*Lilly*, the astrologer, his license to practise physic 129

Lettuce, the garden, its soporific property 130

Liquor ferri alkalina, its properties 142

London, on the healthiness of 195

*Liquor Ammoniæ*, mistakes of the College in its preparation and dose 223, 9

*Liquor Arsenicalis*, remarks on the College direction for preparing 231

Lymph, analysis of 390

London and Paris, description of the medical schools at 461

——, opportunities for acquiring professional knowledge in 464

Lymph in the ventricles of the brain, analysis of 472

LECTURES, by Mr. Brooks, Dr. Reid, Dr. Merriman, Dr. Clutterbuck, Dr. Clough, Dr. Ramsbotham, at St. Thomas and Guy's Hospitals, by Mr. Moor, Dr. Buxton, Mr. Armiger, Mr. Carpue, Dr. Clarke, 242, 3, 4. At the Glasgow Medical School, 338. By Dr. Adams, 338. Drs. Hooper and Agar, Mr. Carlisle, Mr. Brooks, Dr. Squires, Dr. Ramsbotham, 339. Dr. George Pearson, 340. On Anatomy, by Mr. Armiger, 425. By Dr. Adams and Mr. Stevenson, 517. By Dr. Clough and Mr. Singer, 518.

MEDICINE, report on the progress of 1

————, domestic, futility of treatises on 18

Magnesia in calculus, observations on 36

Mineral substances filling the fissures in the earth, observations on 75

Man-midwife, origin of the term 10[illegible]

Medical reform, remarks on 107
METEOROLOGICAL TABLES 166, 167, 255, 343, 430, 519
Murray, Mr. on small-pox and vaccination 180
Medicine-act, new, explanatory remarks on 233
Mineralogy of St. David's 236
Medicine and surgery papers in the Phil. Trans. on 324
Matter, essential properties of, with relation to vitality 330
———, elementary properties of 332
Medical reform, remarks on 383
Miller, Dr. Edward, New York, death of 424
Mania and nervous diseases, Trait distinguishing them 444
Medical practice, instances of the facility of entering on, in England 465
Man, varieties of, arise from climate asserted 489
————— controverted on Humboldt's facts 490
Mercurial action, on the diseases supposed to be produced by 508
Notice to correspondents 344, 432, 520
Nutmeg proved to have no taste 459
Ostrich, analysis of its urine 470
Ovarium, fœtus extracted from 516
Prostate gland, middle lobe of, discovered 6
Purging, observations on employing, in typhus 102
*Poison*, remarkable effects of a sedative, by Dr. Clarke 173
———, supposed vegetable, history of its effects 182
*Pigot*, Dr. on the utility of purging in typhus 289
Petrifaction, observations on the process of 304
Population of Great Britain 307
————— various places 309
Phil. Trans. account of their course of publication 315
—————, account of the papers in 317
Physiology, account of the papers on, in the Phil. Trans. 322
Purgatives, on their use, by Dr. Dickson 446
————————— in yellow fever 447
*Pinckard*, Dr. case of hydrophobia 449
Quickening, short remarks on 41
Queries to correspondents 432
——— on bangue, syphilis, and artificial noses of India 487, 488
Report of the progress of medicine 1
Reform, medical, remarks on 107
Register of the epidemic constitution, from April 1810, to March 1811 137
Report of the Board of Health at New York, 1811 144
Rocks containing rock-salt and coal, account of 157
*Rain*, monthly quantity of 166, 167, 255, 430, 519
*Roget*, Dr. reply to Mr. Hume, on a test for arsenic 187
Regulations for the study of surgery 311
Royal Society, establishment and history of 314
*Roget*, Dr. on Mr. Hume's test for arsenic 469
Stomach, state of, in rabies 16
*Saunders*, *Cunningham*, short biography of 19, nota.
*Spalding*, Dr. bills of mortality for Portsmouth 82
Sponges, essay on 75
Sublimate, corrosive, experiments with 126
*Spina bifida*, on the treatment of, by Ashley Cooper 153
Society, new, Literary and Phil. at Liverpool 162
Sol-lunar influence in a case of catalepsy 169
Sand drifted, remarkable tubes found in 239
*Stevenson*, Mr. on cataract 257, 358
*Saunders*, Mr. letter on the operation for cataract 258
Scurvy, observations on 270, 1, 2, 3
Surgery, regulations for the study of, at St. George's 311
Small-pox, deaths by, in London, in August and July 340
Serum of the blood, remarks on, analysis of 391
Society, Phil. of London, meeting of, 422, 512—Medical, of London—Blenheim-street—Westminster—Linnæan, meetings of, 424.
Small pox, deaths by, in London, from the 8th of September to the 6th of October 425
*Speer*, Dr. T. C. on the physical constitution of water 433
Sensation, morbid, Dr. Kinglake on 441
—————————, case of 443
Small-pox, secondary case of 456
Smell and taste, observations on the senses of 457
Schools, medical, of London and Paris compared 461

### INDEX.

Syphilis, a cure for, without mercury 475
———, on its existence in Otaheite 494
Travers, Mr. on wounds of the intestines 8
*Taunton*, Mr. calculation on the frequency of hernia 40
Typhus, observations on purging in 102
Twin-cases, observations on 104
Teneriffe, account of 236
Typhus, on purgatives in 278
*Thompson*, Dr. reply to Mr. *Fogo* 279
Tanning of leather, by pyrolignous or wood acid, patent for, by Mr. Patten 427
*Teale*, Mr. secondary case of small-pox 456
Taste and smell, observations on the senses of 457
———, nutmeg proved to have none 459
Tuition, medical, in London and Paris, compared 461
Tincture, anti-syphilitic, of M. Besnard, composition of 473
Typhus, on blood-letting in 476
Tubercle, subcutaneous, history of that remarkable disease 506
Urine, analysis of that of the ostrich 470
———, of the rose-colored sediment in 471
Vaccination, a new method to prevent mistakes in 159
——— in Jamaica, Mr. Foot on 78
——— and small-pox, remarks on, by Mr. Murray 180
Vaccine establishment national, report of, 1812 190
Volcano off St. Michael's, narrative of its eruption 200
Vitrified fort, account of 240
Vaccine inoculation, success and progress of 242
Vapor-bath medicated 242
Vermes, intestinal, on 413
Vaccine vesicle, retarded appearance of 452
Water, mineral, in the Isle of Wight, discovered 22
Wounds, on the fatal termination of, in the navy 89
———, the probable cause of 91
———, method of preventing 92
*Woorara*, experiments with 116
Wax, a vegetable, account of 136
Wind of a ball, observations on 142
Water, inquiry into the physical constitution of, by Dr. Speer 433
———, fluidity of, observations on various theories of 434
*Willan*, Dr. Robert, biographical memoir of 477
———, commencement of his series of medical reports in the Monthly Magazine 484
———, on medical physiognomy 487
York, New, report of the Board of Health at, 1811 144
Zoology, remarks on 321

PLATES.

Hernia Cerebri - - - - - - - - Page 186
Mr. Stevenson's Cataract, Needle, and Knife - - 257
Mr. Jukes' unusual Conformation of Arteries - - 440

